# Integrative Transcriptomic, Proteomic and Functional Analysis Reveals ATP1B3 as a Diagnostic and Potential Therapeutic Target in Hepatocellular Carcinoma

**DOI:** 10.3389/fimmu.2021.636614

**Published:** 2021-04-02

**Authors:** Shanshan Lu, Shenglan Cai, Xiaozhen Peng, Ruochan Cheng, Yiya Zhang

**Affiliations:** ^1^ National Clinical Research Center for Geriatric Disorders, Xiangya Hospital, Central South University, Changsha, China; ^2^ Research Center of Carcinogenesis and Targeted Therapy, Xiangya Hospital, Central South University, Changsha, China; ^3^ The Higher Educational Key Laboratory for Cancer Proteomics and Translational Medicine of Hunan Province, Xiangya Hospital, Central South University, Changsha, China; ^4^ Hunan Key Laboratory of Viral Hepatitis, Xiangya Hospital, Central South University, Changsha, China; ^5^ Huaihua Key Laboratory of Research and Application of Novel Molecular Diagnostic Techniques, School of Public Health & Laboratory Medicine, Hunan University of Medicine, Huaihua, China; ^6^ Department of Hunan key laboratary of aging biology, Xiangya Hospital, Central South University, Changsha, China; ^7^ Department of Dermatology, Xiangya Hospital, Central South University, Changsha, China

**Keywords:** Na^+^/K^+^-ATPase (NKA), hepatocellular carcinoma (HCC), ATP1B3, biomarker, immune

## Abstract

The Na+/K+-ATPase (NKA), has been proposed as a signal transducer involving various pathobiological processes, including tumorigenesis. However, the clinical relevance of NKA in hepatocellular carcinoma (HCC) has not been well studied. This study revealed the upregulation of mRNA of ATP1A1, ATP1B1, and ATP1B3 in HCC using TCGA, ICGC, and GEO database. Subsequently, ATP1B3 was demonstrated as an independent prognostic factor of overall survival (OS) of HCC. To investigate the potential mechanisms of ATP1B3 in HCC, we analyzed the co-expression network using LinkedOmics and found that ATP1B3 co-expressed genes were associated with immune-related biological processes. Furthermore, we found that ATP1B3 was correlated immune cell infiltration and immune-related cytokines expression in HCC. The protein level of ATP1B3 was also validated as a prognostic significance and was correlated with immune infiltration in HCC using two proteomics datasets. Finally, functional analysis revealed that ATP1B3 was increased in HCC cells and tissues, silenced ATP1B3 repressed HCC cell proliferation, migration, and promoted HCC cell apoptosis and epithelial to mesenchymal transition (EMT). In conclusion, these findings proved that ATP1B3 could be an oncogene and it was demonstrated as an independent prognostic factor and correlated with immune infiltration in HCC, revealing new insights into the prognostic role and potential immune regulation of ATP1B3 in HCC progression and provide a novel possible therapeutic strategy for HCC.

## Introduction

Hepatocellular carcinoma (HCC) is a primary liver cancer with high mortality and is the most common malignancy (1), which occurs frequently in Asia, Africa, southern Europe and China (2). Although early surgical resection and liver transplantation are effective treatments for HCC (3), the 5-year recurrence rate for HCC remains poor because of its high recurrence and metastasis rates (4). Therefore, useful prognostic and therapeutic indicators are urgently needed.

The ion transporter Na^+^/K^+^-ATPase (NKA) is a transmembrane protein that transports Na^+^ and K^+^ across cell membranes (5), which is essential for the cellular electrochemical gradient (6), ion homeostasis (7), cell adhesion (8), and intracellular signaling (9). The functional NKA consists of α subunits and β subunits. So far, 4 NKA α-subunits (α1, α2, α3, and α4) and 4 β-subunits (β1, β2, β3, and β4) have been identified. The abnormal NKA could lead to a variety of diseases, including hypokalaemic periodic paralysis and CNS symptoms (10), cardiovascular disorders (11), atherosclerosis (12), Alzheimer (13). Recent studies showed that NKA was dysregulated in multiple cancers and involved in the progression of these cancers (14). For example, Mathieu et al. (15) showed that the NKA α1 subunit is highly expressed in human melanoma and involved in cell migration and apoptosis. Lee et al. (16) reported that the NKA β1 subunit is low-expressed in medulloblastoma. Bechmann et al. (17) revealed that NKA α1, α3, and β1 subunits were highly expressed in colorectal cancers and associated with tumor metastases. Nevertheless, the clinical relevance of NKA in HCC remains unclear.

In this study, we investigated the expression of NKA α/β subunits in HCC using 6 independent public datasets. We demonstrated ATP1B3 as a prognostic factor which is correlated with immune infiltrating in HCC. Functional analysis revealed ATP1B3 as a potential oncogene of HCC, indicating that ATP1B3 as a diagnostic and potential therapeutic target in HCC.

## Materials and Methods

### NKA Expression in Different Datasets

The expression levels of NKA α/β subunits in HCC were identified from ICGC (https://icgc.org/daco) and TCGA (https://cancergenome.nih.gov/) datasets (18). Then, the expression levels of ATP1A1, ATP1B1, and ATP1B3 were verified in three independent GEO datasets (GSE45436, GSE76427 and GSE102079) download from https://www.ncbi.nlm.nih.gov/gds (19).

The transcription levels of NKA genes in various cancers were detected in the GEPIA database (http://gepia.cancer-pku.cn/) (20) and ONCOMINE database (https://www.oncomine.org/) (21). The thresholds were set as: logFC > 1 and *p* < 0.01.

### Survival Analysis

The prognostic value of ATP1A1, ATP1B1, and ATP1B3 for HCC in the TCGA database were appraised by the Kaplan-Meier plotter database (http://kmplot.com/analysis/) (22) and then validated using the ICGC database using R software (version 3.5.2).

### The Relationship Between ATP1B3 and Clinical Characteristics of HCC

The expression of ATP1B3 in HCC patients with different clinical characteristics was analyzed using R software and then validated using the UALCAN database (http://ualcan.path.uab.edu) (23). The significance of differential gene expression was assessed by t-test and one-way ANOVA. *, *p* < 0.05; **, *p* < 0.01; ***, *p* < 0.001.

### LinkedOmics Database Analysis

The co-expressed genes of ATP1B3 in HCC was detected using LinkedOmics (http://www.linkedomics.org/login.php). Co-expressed genes can be analyzed statistically and displayed in the volcano, Heat maps. Gene set enrichment analysis (GSEA) can also be used in LinkedOmics functional modules to perform Gene Ontology (GO) term annotation, KEGG pathway analysis, and target enrichment of kinases, miRNAs, and transcription factors’ (TF) (24). Pearson test was used to evaluate the significant correlation of co-expressed genes. FDR < 0.01 was significant expression, *p* < 0.05 was significantly related genes.

### Correlations of ATP1B3 Expression With Immune Infiltration in TIMER and GEPIA

The association between ATP1B3 and immune cells infiltration in HCC was confirmed using the TIMER database (http://cistrome.org/TIMER/). It provides the infiltration of 6 types of immune cells to assess the abundance of immune infiltration (25, 26). Furthermore, the expression of ATP1B3 in immune subtypes and molecular subtypes in HCC was identified using the TISIDB database (http://cis.hku.hk/TISIDB/). It integrates a large amount of tumor immunity-related data, including 988 genes related to anti-tumor immunity, and can analyze the data of 30 TCGA cancer types to calculate the gene expression of immune subtypes and molecular subtypes in different tumors (27).

Next, the correlations between ATP1B3 and immune markers expression in HCC was investigated using the TIMER and GEPIA databases. These immune markers have been referenced previously (28). The correlation between ATP1B3 and each immune gene markers was presented using scatterplots, Pearson test was used for statistical significance evaluation, and log2 RSEM was adopted to regulate gene expression levels. ATP1B3 was plotted on the y-axis, while marker genes are plotted on the x-axis.

### Proteomics Database Analysis

The expression and prognosis of ATP1B3 protein were generated using the CPTAC proteomics database (https://cptac-data-portal.georgetown.edu/cptacPublic/). Moreover, the proteomics and phospho-proteomics data from 316 HCC patients were download from Gao’s work (29). These data can well verify the relationship between proteins and survival and clinical, and find candidate proteins that can be used as tumor biomarkers (30, 31).

### Cell Lines and Culture

HCC cell lines (Huh7 and HCCLM3) and human normal liver cell (LO2) were obtained from American Type Culture Collection (ATCC, Manassas, VA, USA). Huh7 and HCCLM3 were cultured in RPMI-1640 medium (BI, Israel) containing 10% FBS (BI, Israel) at 37° C in 5% CO_2_. And LO2 cultured in DMEM (BI, Israel) medium containing 10% FBS (BI, Israel) at 37° C in 5% CO_2_.

### qRT-PCR and Western Blot

The protein and mRNA expression levels of ATP1B3 in the HCC cells and normal liver cell were detected by Western blots and qRT-PCR, respectively, as described previously by us (32–34). The Anti-ATP1B3 antibody was purchased from Santa (sc-135998, 1:50), the Anti-Tubulin antibody was purchased from Elabscience (E-AB-20036, 1:2000), and the ATP1B3 primer used for the amplification was as follows: 5′-TGATCCAACTTCGTATGCAGGG-3′ and 5′-ACATGCAACATAAACTGGACCC-3′ (Sangon Biotech, China).

### Transfection of siATP1B3

50 nM of siATP1B3 was transfected into HCC cells by using Lipofectamine™ 2000 (Invitrogen) according to the manufacturer’s instruction (35). The ATP1B3 siRNA was 5′-CUCAUAAUGGAAUGAUAGATT-3′ and 5′-UCUAUCAUUCCAUUAUGAGTT-3′ (TSINGKE, China).

### Cell Migration Assay

Transwell migration assay and wound healing assay were performed as described previously (36, 37). The assay was performed three times in triplicate.

### Plate Clone Formation and MTT Assay

Cell proliferation was monitored by Plate clone formation and MTT assay as described previously by us (38, 39). The assay was performed three times in triplicate.

### Cell-Cycle and Cell Apoptosis Assay

Cell-cycle and cell apoptosis were performed by flow cytometry analysis as described previously (35, 40). The assay was performed three times in triplicate.

### Clinical Samples and Immunohistochemistry (IHC)

Fifteen formalin-fixed, paraffin-embedded HCC and paired adjacent liver tissues were collected from Xiangya Hospital of Central South University from September 2019 to January 2020. Our study was approved by the ethics committee of Xiangya Hospital, Central South University.

According to our previously described (41, 42), IHC and an immunoreactive score of ATP1B3 (Anti-ATP1B3 antibody: 67554-1-Ig, proteintech, 1:1000) were conducted on the formalin-fixed and paraffin-embedded tissue sections.

### Statistical Analysis

Statistical obtained from TCGA were all analyzed by R-3.6.1. The differential expression of the 8 NKA genes in the TCGA and ICGC cohort were evaluated using the “limma” and “vioplot” package, and the heat map was generated using the heatmap package of the R software. The survival package was used for the survival analysis of the sample from ICGC. The relationship of ATP1B3 expression and clinical characteristics were assessed applying logistic regression. Univariate and multivariate analysis revealed the relationship between ATP1B3 and the clinical factors, the immune cell infiltration with OS of HCC using the “survival” R package. The ROC curves, with AUC values quantified with the survival ROC package. Other data were calculated statistically using SPSS software ver20.0 (SPSS, Inc, Chicago, IL, USA) and GraphPad Prism 7.0 (GraphPad Software, La Jolla, CA, USA). *p* < 0.05 was considered statistically significant.

## Results

### NKA Genes Expression in HCC

**Figure 1:**
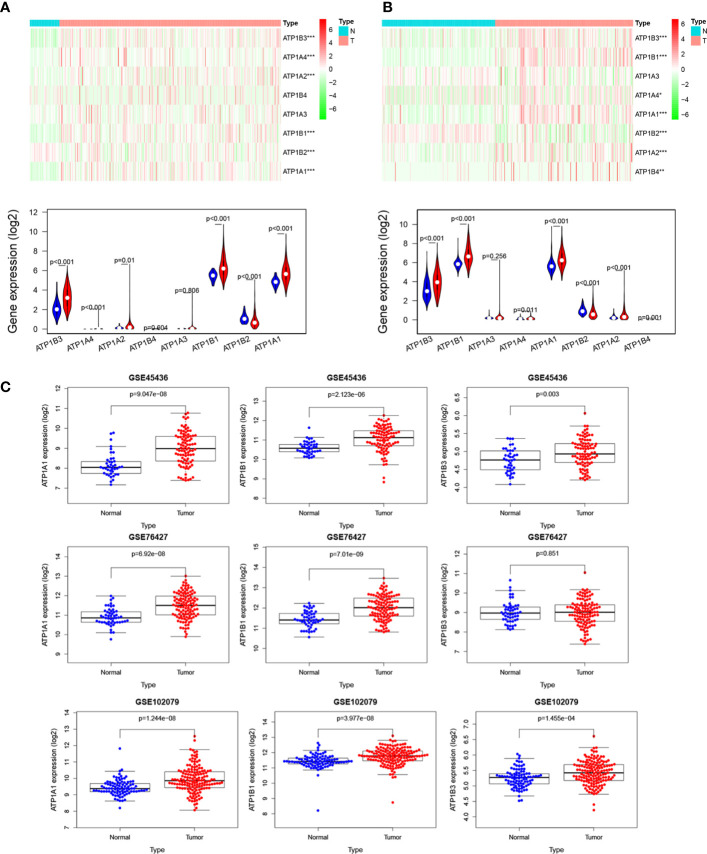
NKA genes expression in HCC. The mRNA levels of NKA genes in HCC from **(A)** TCGA database. Up, heatmap. Down, Violin plot, Red: HCC tissue; Blue: normal tissue. **(B)** ICGC database. Up, heatmap. Down, Violin plot, Red: HCC tissue; Blue: normal tissue. **(C)** The mRNA levels of ATP1A1, ATP1B1, and ATP1B3 in HCC from four independent GEO datasets (GSE45436, GSE76427, GSE64041, and GSE102079).

We first analyzed the mRNA level of 8 NKA genes (ATP1A1-4, ATP1B1-4) in HCC using TCGA and ICGC (LIRI-JP) datasets. Among these, the mRNA expression of ATP1A1, ATP1B1, and ATP1B3 were evidently increased in HCC compared to normal tissue in TCGA with logFC >1 and p<0.01 (**Figure 1A** and **Table S1**). Although ATP1A2, ATP1A4, and ATP1B2 were differently expressed between HCC tissue and normal tissue, ATP1A2, ATP1B2 and ATP1A4 mRNA levels were very much low in both HCC and normal tissue, and ATP1B2 expression was slightly reduced in HCC compared to liver tissue with |log2FC|<0.5. Similar results were also observed in ICGC (LIRI-JP) datasets (**Figure 1B** and **Table S2**). Subsequently, the expression levels of three genes (namely ATP1A1, ATP1B1, and ATP1B3) were also validated in 3 independent GEO datasets (GSE45436, GSE76427, and GSE102079) (**Figure 1C**). Finally, the Oncomine database and GEPIA database showed that the mRNA expression of ATP1B1 and ATP1B3 are widely upregulated in various cancers, including Leukemia, Lung cancer, Lymphoma, Head and neck cancer and so on (**Figures S1**–**S3**).

### Prognostic Value of ATP1A1, ATP1B1, and ATP1B3 in HCC

**Figure 2:**
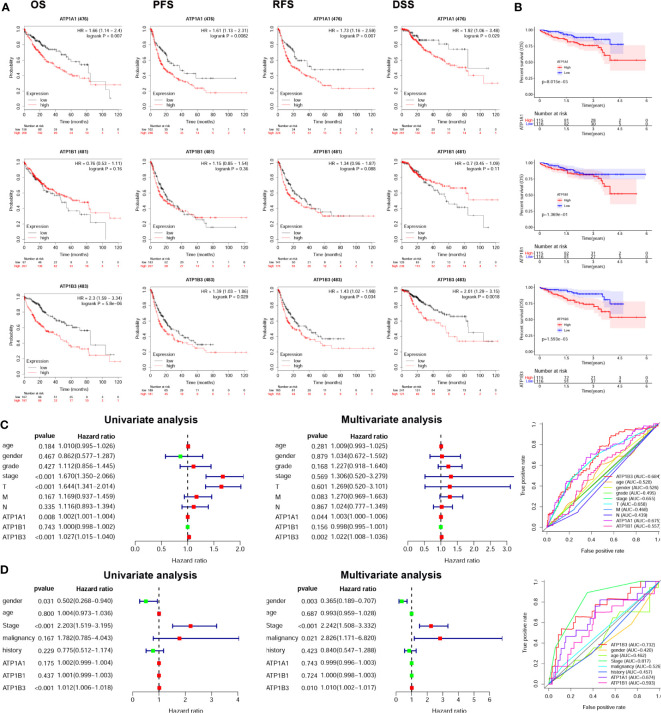
ATP1A1, ATP1B1, and ATP1B3 mRNA are associated with prognosis of HCC patients. **(A)** The survival curves of OS, PFS, RFS, and DSS with high/low ATP1A1, ATP1B1 and ATP1B3 in TCGA HCC cohorts using the Kaplan-Meier plotter (OS, n=364; RFS, n=316; PFS, n=370; DSS, n=362). The high and low mRNA expression is splitting by best cutoff. **(B)** The survival curves of OS with high/low ATP1A1, ATP1B1, and ATP1B3 in ICGC HCC cohorts, the high and low mRNA expression is splitting by median. Univariate and multivariate analysis and ROC curve revealed the relationship between ATP1A1, ATP1B1, ATP1B3, and the clinical factors with overall survival of HCC in **(C)** TCGA database and **(D)** ICGC database. (T, stage T; N, stage N; M, stage M).

We next investigated the prognostic value of ATP1A1, ATP1B1, and ATP1B3 for HCC using the Kaplan-Meier plotter. The HCC patients with high ATP1A1 showed worse overall Survival (OS: HR = 1.66 (1.14-2.4), *p* = 0.007), Progression-Free Survival (PFS: HR = 1.61 (1.13-2.31), *p* = 0.0082), Relapse Free Survival (RFS: HR = 1.73 (1.16-2.59), *p* = 0.007) and Disease Free Survival (DSS: HR = 1.92 (1.06-3.48), *p* = 0.029) in **Figure 2A**. High ATP1B3 was related to worse prognosis in HCC (OS: HR = 2.3 (1.59-3.34), *p* = 5.8E-6; PFS: HR = 1.39 (1.03-1.86), *p* = 0.029; RFS: HR = 1.43 (1.02-1.98), *p* = 0.034; DSS: HR = 2.01 (1.29-3.15), *p* = 0.0018). Similar results were also observed in the ICGC database (**Figure 2B**). Moreover, the univariate and multivariate analysis showed that only ATP1B3 was an independent prognostic factor for OS of HCC using both TCGA and ICGC database (**Figures 2C, D**). Finally, the AUC values of ATP1B3 for the OS model from TCGA and ICGC database were 0.684 and 0.732 respectively, which were more sensitivity and specificity than the clinical factors (**Figures 2C, D**, right). These results indicated that ATP1B3 was an independent prognostic biomarker for HCC.

### ATP1B3 Is Correlated With Clinicopathological Characteristics in HCC

**Figure 3:**
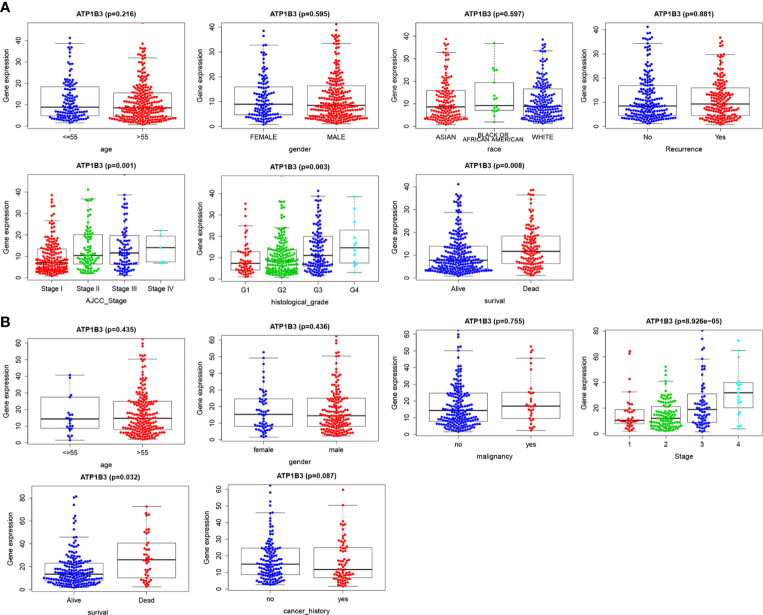
ATP1B3 is correlated with clinicopathological characteristics of HCC patients. **(A)** TCGA database. **(B)** ICGC database.

Based on the clinical data extracted from TCGA-LIHC, we found that high ATP1B3 was associated with higher stage, higher grade, and more dead (p=0.01, p=0.03, and p=0.008) (**Figure 3A**). Consistent with these results, high ATP1B3 was associated with higher stage, higher grade, and more dead in the ICGC database (**Figure 3B**). These results were also confirmed by the UALCAN (**Figure S4**).

We next explored the association between ATP1B3 expression and the clinicopathological characteristics of HCC patients using Kaplan-Meier Plotter (**Table 1**). For OS, high expressed ATP1B3 related with poor OS in all stage, grade I/II/III, T 1/2/3, none-vascular invasion, grade (male/female), White and Asian race, no-Alcohol consumption, both with or without Hepatitis virus. For PFS, ATP1B3 expression was significantly hazardous to HCC patients with stage I, grade II, T 1, none-vascular invasion, female, Asian, and Hepatitis virus (**Table 1**).

**Table 1:** Correlation of ATP1B3 mRNA expression with OS (*n* = 364) and PFS (*n* = 370) in liver hepatocellular carcinoma with different clinicopathological features.

OS (364)	Number	HR	*p* value	PSF (370)	Number	HR	*p* value
Stage				Stage			
I	170	2.58 (1.4-4.77)	0.0017	I	170	1.84(1.08-3.14)	0.0225
I+II	253	2.32(1.44-3.75)	0.0004	I+II	254	1.4(0.91-2.13)	0.1201
II	83	2.4(0.9-6.38)	0.0706	II	86	0.65(0.36-1.17)	0.1469
II+III	166	2.1(1.25-3.53)	0.0043	II+III	167	0.81(0.54-1.21)	0.3001
III	83	2.44(1.27-4.67)	0.0056	III	83	1.45(0.82-2.54)	0.1968
III+IV	87	2.54(1.34-4.83)	0.0032	III+IV	88	1.39(0.8-2.4)	0.242
IV	4	–	–	IV	5	–	–
Grade				Grade			
I	55	0.67(0.25-1.77)	0.4166	I	55	1.65(0.73-3.73)	0.2218
II	174	2.72(1.57-4.72)	0.0002	II	175	1.83(1.18-2.85)	0.0063
III	118	2.9(1.58-5.34)	0.0003	III	119	1.42(0.86-2.34)	0.1682
IV	12	–	–	IV	12	–	–
AJCC_T				AJCC_T			
I	180	2.43(1.35-4.38)	0.0023	I	180	1.7591.04-2.93)	0.0329
II	90	2.66(1.02-6.99)	0.0384	II	92	0.68(0.38-1.21)	0.1827
III	78	2.65(1.21-5.8)	0.0116	III	78	1.48(0.77-2.84)	0.2418
IV	13	–	–	IV	13	–	–
Vascular invasion				Vascular invasion			
none	203	2.73(1.62-4.61)	8.90E-05	none	204	1.86(1.19-2.9)	0.0058
micro	90	1.9(0.82-4.37)	0.1261	micro	91	0.59(0.3-1.12)	0.1013
macro	16	–	–	macro	16	–	–
Gender				Gender			
male	246	3.33(1.98-5.58)	1.40E-06	male	246	1.35(0.93-1.96)	0.1113
female	118	2.49(1.17-5.32)	0.0146	female	120	1.75(1.05-2.94)	0.0312
Race				Race			
white	181	1.62(0.99-2.64)	0.0517	white	183	1.41(0.94-2.12)	0.0914
black or african	17	–	–	black or african	17	–	–
asian	155	4.38(2.1-9.12)	1.70E-05	asian	155	1.81(1.13-2.91)	0.0129
Alcohol consumption				Alcohol consumption			
yes	115	1.78(0.9-3.51)	0.0936	yes	115	1.46(0.87-2.47)	0.1536
no	202	2.4(1.52-3.78)	0.0001	no	204	1.54(0.98-2.44)	0.0613
Hepatitis virus				Hepatitis virus			
yes	150	2.68(1.32-5.42)	0.0045	yes	152	3.2(1.54-6.64)	0.0009
no	167	189(1.15-3.11)	0.0109	no	167	1.49(0.91-2.43)	0.11

Short bars appear due to limited sample size for parameters and hazard ratio cannot be calculated. OS, overall survival; PFS, progression-free survival. *, p < 0.05.

### ATP1B3 Co-Expression Networks in HCC

**Figure 4:**
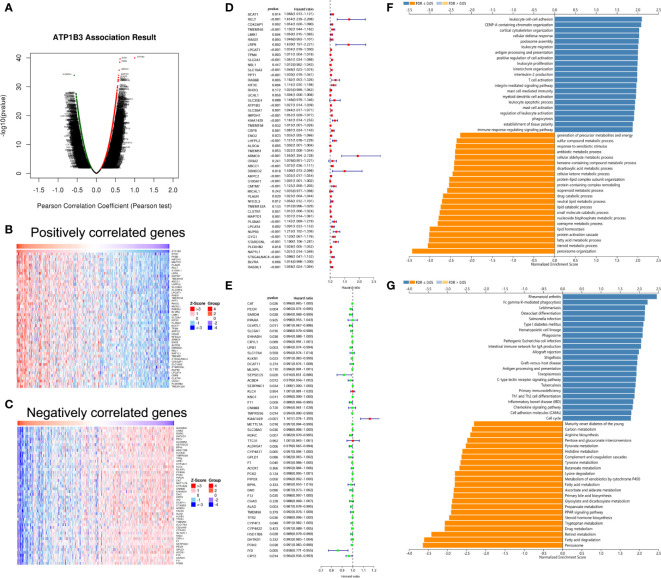
ATP1B3 co-expression genes in HCC using the LinkedOmics. **(A)** The volcano plot of ATP1B3 co-expression genes. Heat maps revealed the top 50 positively **(B)** and negatively **(C)** co-expressed genes of ATP1B3 in HCC. Cox analysis revealed the prognosis of 50 positively **(D)** and negatively **(E)** co-expressed genes of ATP1B3 in HCC. The GO enrichment **(F)** and KEGG pathways **(G)** of ATP1B3.

We analyzed the ATP1B3 co-expression networks in HCC using LinkedOmics. As shown in **Figure 4A**, a total of 9,531 genes expression were significant correlations with ATP1B3 expression (FDR < 0.01) with 2,564 (green dots) negatively correlated genes and 6,967 positively correlated genes (red dots). The top 50 positively and negatively co-expressed genes were shown in the heat map (**Figures 4B, C** and **Table S3**). Among these, 39 of 50 positive genes and 21 of 50 negative genes were associated with OS of HCC with a high/low hazard ratio (HR) (*p* < 0.05) (**Figures 4D, E**).

GO annotation revealed that these genes participate in various immune response, including leukocyte cell-cell adhesion, leukocyte migration, antigen processing and presentation, leukocyte proliferation. In contrast, various metabolic processes were inhibited, including steroid metabolic process, antibiotic metabolic process, fatty acid metabolic process, and dicarboxylic acid metabolic process (**Figure 4F** and **Table S4**). KEGG pathway analysis showed the enrichment in immune and metabolic pathways, including rheumatoid arthritis, Fc gamma R-mediated phagocytosis, Leishmaniasis, and so on (**Figure 4G** and **Table S5**). These findings demonstrated that ATP1B3 is involved in the immune response and the metabolic regulation of HCC.

### ATP1B3-Related Networks in HCC

To address the ATP1B3-related network in HCC, we analyzed the transcription factors (TF), miRNAs, and kinases in ATP1B3 co-expressed genes. The top 3 most significant related kinases are LCK proto-oncogene (LCK), p21 (RAC1) activated kinase 1 (PAK1), LYN proto-oncogene (LYN) (**Tables 2** and **S6**). No ATP1B3 co-expressed miRNA was enriched by GSEA (**Table S7**). The most significant ATP1B3 co-expressed TF was belong to the SRF transcription factor family **(Table S8)**, including CFL1, CAP1, SUSD1, FOSL1, KCNMB1.

**Table 2:** The Kinases, miRNAs and transcription factors-target networks of ATP1B3 in HCC.

Enriched Category	Geneset	Leading Edge Number	*p* Value	FDR
miRNA_target	GCAAGAC,MIR-431	22	0	0.13008
	ACACTCC,MIR-122A	36	0.002242	0.15456
	GGGGCCC,MIR-296	33	0.00231	0.19513
	AGGAAGC,MIR-516-3P	32	0.002151	0.21191
	GCGCTTT,MIR-518B,MIR-518C,MIR-518D	8	0.057221	0.26356
	TACGGGT,MIR-99A,MIR-100,MIR-99B	13	0.048223	0.27066
	GGCCAGT,MIR-193A,MIR-193B	33	0.018182	0.2822
	AACTGAC,MIR-223	23	0.017978	0.28409
	GTGGTGA,MIR-197	32	0.02069	0.29278
	GTGTGAG,MIR-342	31	0.021327	0.30936
Transcription_Factor_target	V$SRF_01	25	0	0.003348
	V$HNF4_01	73	0	0.004539
	RGAGGAARY_V$PU1_Q6	218	0	0.005022
	V$ELF1_Q6	92	0	0.006428
	V$PEA3_Q6	110	0	0.00703
	GGGNNTTTCC_V$NFKB_Q6_01	60	0	0.008035
	RGTTAMWNATT_V$HNF1_01	23	0	0.009078
	V$CP2_02	121	0	0.011048
	V$AP1_Q6_01	95	0	0.011717
	V$HNF1_01	57	0	0.012347
Kinase_target	Kinase_LCK	26	0	0.038989
	Kinase_PAK1	21	0	0.041155
	Kinase_LYN	30	0	0.049819
	Kinase_PRKCB	37	0	0.05361
	Kinase_ITK	5	0.010959	0.070999
	Kinase_PRKG1	13	0.005076	0.073375
	Kinase_PRKCG	16	0.002268	0.078845
	Kinase_SYK	19	0	0.081382
	Kinase_ROCK1	17	0	0.092708
	Kinase_PLK1	45	0	0.094946

*p < 0.05, **p < 0.01, ***p < 0.001

### The Association of ATP1B3 and Immune Infiltration in HCC

**Figure 5:**
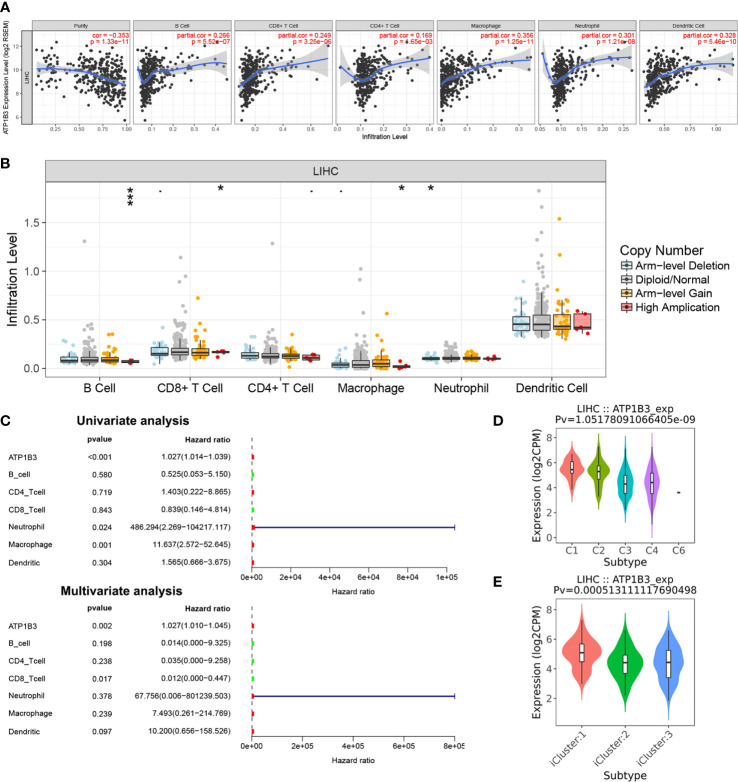
The association of ATP1B3 and immune infiltration level in HCC using the TIMER. **(A)** The correlation between ATP1B3 expression level and immune infiltration. **(B)** The relationship between ATP1B3 CNV and immune infiltration. *p < 0.05, **p < 0.01, ***p < 0.001. **(C)** The prognosis of ATP1B3 and immune cells for OS of HCC. **(D)** Associations between ATP1B3 expression and immune subtypes in HCC. C1 (wound healing); C2 (IFN-gamma dominant); C3 (inflammatory); C4 (lymphocyte depleted); C5 (immunologically quiet); C6 (TGF-b dominant). **(E)** Associations between ATP1B3 expression and molecular subtypes in HCC.

Basing on the GO analysis, we next detected the correlations of ATP1B3 and immune cells in HCC using the TIMER. We found that ATP1B3 was correlated with tumor purity (r = -0.353, *p* = 1.33E-11) and the B cells infiltration (r =0.266, *p* = 5.52E-7), CD8+ T infiltration (r = 0.249, *p* = 3.25E-6), CD4+ T infiltration (r = 0.169, p = 1.65E-3), Macrophage infiltration (r = 0.356, *p* = 1.25E-11), Neutrophil infiltration(r = 0.301, *p* = 1.21E-8) and Dendritic cell infiltration (r = 0.328, *p* = 5.46E-10) (**Figure 5**). Particularly, ATP1B3 CNV has evidently correlated with immune infiltration including B cells, CD8+ T cells, macrophages and neutrophils (**Figure 5**). Moreover, Univariate analysis showed that ATP1B3, Neutrophil and Macrophage were significantly associated with OS in HCC, and multivariate analysis showed that ATP1B3 and CD8+ T cells were independent factors of OS in HCC (**Figure 5**). Furthermore, ATP1B3 was also observed differently expressed in immune subtypes (**Figure 5**) and molecular subtypes (**Figure 5**) in HCC using TISIDB database. In addition, **Figure S5** showed that ATP1B3 was associated immune cells infiltration in pan-cancer.

### The Correlation Between ATP1B3 and Immune Markers and Immune-Related Cytokines in HCC

**Figure 6:**
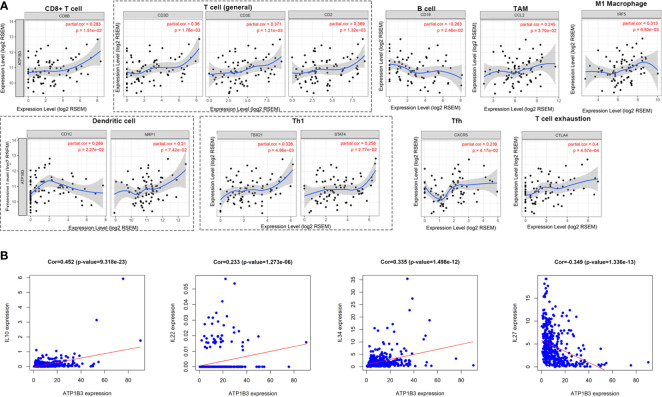
Correlation between ATP1B3 and immune markers and immune-related cytokines in HCC. **(A)** The correlation between ATP1B3 and immune-related marker genes in HCC (TIMER). **(B)** The correlation between ATP1B3 and HCC-related cytokines.

Next, we investigated the ATP1B3 crosstalk with immune cells, basing on the correlations between ATP1B3 and immune-related gene expression in HCC using the TIMER (**Figure 6** and **Table 3**) and GEPIA databases (**Table 4**). The results revealed that ATP1B3 expression was positively correlated with the makers of CD8+ T, T cell, M1 Macrophage, B cell, TAM (tumor-associated macrophage), DCs, Th1 (T helper cell 1), Tfh (Follicular helper T cell), and T cell exhaustion. Moreover, ATP1B3 was also associated with HCC-related cytokines and chemokines. Our research shows that the expression of ATP1B3 is positively correlated with IL10, IL22, IL34 and negatively correlated with IL27 (**Figure 6B**). These findings revealed the potential association between ATP1B3 and immune cell infiltration in HCC. As HCC is associated a higher level of inflammation, it is relatively evident that every marker upregulated to such HCC initiation and progression will be correlated to inflammation markers. So further experiments are needed for this speculation.

**Table 3:** Correlation analysis between ATP1B3 and relate genes and markers of immune cells in TIMER.

Description	Gene markers	HCC			
		None		Purity	
		Core	*p*	Core	*p*
CD8+ T cell	CD8A	0.193695	0.087216	0.226704	0.053766
	CD8B	0.24628	0.028676	0.283328	0.015142
T cell (general)	CD3D	0.295098	0.008287	0.360033	0.001756
	CD3E	0.30925	0.005732	0.371476	0.001213
	CD2	0.29944	0.007545	0.368986	0.001316
B cell	CD19	-0.21158	0.06123	-0.2629	0.024633
	CD79A	0.206353	0.068175	0.190102	0.107196
	CD20/KRT20	0.069475	0.542921	0.107764	0.364148
	CD38	0.17554	0.121766	0.178714	0.130333
Monocyte	CD86	0.219279	0.052356	0.224556	0.056138
	CD115/CSF1R	0.193744	0.087135	0.219431	0.062145
TAM	CCL2	0.213121	0.059468	0.244564	0.037045
	CD68	0.123539	0.277431	0.081046	0.495473
	IL10	0.08718	0.44488	0.082802	0.486149
M1 Macrophage	iNOS/NOS2	0.036588	0.748873	-0.00432	0.971037
	IRF5	0.301388	0.007149	0.313443	0.006929
	COX2/PTGS2	0.178607	0.115283	0.222918	0.058005
M2 Macrophage	CD163	0.080185	0.481597	0.075294	0.526662
	VSIG4	0.097347	0.392623	0.086679	0.465895
	MS4A4A	0.134494	0.236785	0.129154	0.276146
Neutrophils	CD66b/CEACAM8	0.056755	0.619325	0.086414	0.467261
	CD11b/ITGAM	0.165847	0.143872	0.17351	0.142092
	CCR7	0.178271	0.115981	0.174791	0.139127
Natural killer cell	KIR2DL1	0.124381	0.274769	0.089358	0.452167
	KIR2DL3	0.184795	0.103019	0.225681	0.054885
	KIR2DL4	0.124151	0.275663	0.092833	0.434706
	KIR3DL1	-0.13216	0.245613	0.092833	0.434706
	KIR3DL2	0.231732	0.039888	0.213627	0.069564
	KIR3DL3	0.045013	0.693647	0.06811	0.566946
	KIR2DS4	0.190579	0.092498	0.21482	0.067984
Dendritic cell	HLA-DPB1	0.197785	0.08065	0.229444	0.050859
	HLA-DQB1	0.19131	0.091238	0.181263	0.124851
	HLA-DRA	0.126144	0.267366	0.128596	0.278246
	HLA-DPA1	0.154333	0.17412	0.160538	0.174852
	BDCA-1/CD1C	0.292641	0.008867	0.266351	0.022742
	BDCA-4/NRP1	0.205964	0.068706	0.210243	0.074208
	CD11c/ITGAX	0.054771	0.630979	-0.00886	0.94072
Th1	T-bet/TBX21	0.250883	0.025738	0.326148	0.004863
	STAT4	0.230101	0.041346	0.257719	0.027716
	STAT1	0.086246	0.448998	0.063854	0.591474
	IFN-γ/IFNG	0.093085	0.414531	0.142996	0.227475
	TNF-α/TNF	0.175729	0.121358	0.211978	0.071798
Th2	GATA3	0.206938	0.067385	0.200184	0.089491
	STAT6	-0.09837	0.387653	-0.0571	0.631374
	STAT5A	0.161587	0.154563	0.160109	0.17602
	IL13	-0.06835	0.549514	-0.07901	0.506375
Tfh	BCL6	0.14389	0.205385	0.154364	0.19225
	CXCR5	0.208307	0.065442	0.239045	0.041672
	ICOS	0.186286	0.100222	0.20881	0.076248
	BCL-6	0.14389	0.205386	0.154364	0.19225
Th17	STAT3	0.063218	0.579202	0.039726	0.738612
	IL17A	0.180941	0.110532	0.169988	0.150491
Treg	FOXP3	0.040141	0.724896	0.025344	0.831455
	CCR8	0.125941	0.268745	0.082091	0.489913
	STAT5B	0.067259	0.555148	0.060167	0.613102
	TGFβ/TGFB1	0.174513	0.123859	0.161097	0.173335
T cell exhaustion	PD-1/PDCD1	0.182633	0.107183	0.228417	0.051933
	CTLA4	0.365268	0.000933	0.399865	0.000457
	LAG3	0.176874	0.118796	0.16538	0.162032
	TIM-3/HAVCR2	0.060467	0.595846	-0.00268	0.982079
	GZMB	0.196774	0.082188	0.185335	0.11646

HCC: hepatocellular carcinoma; CHOL: cholangiocarcinoma; TAM: tumor-associated macrophage; Th: T helper cell; Tfh: Follicular helper T cell; Treg, regulatory T cell; Cor, R value of Spearman’s correlation; None, correlation without adjustment. Purity, correlation adjusted by purity. *p < 0.05, **p < 0.01, ***p < 0.001.

**Table 4:** Correlation analysis between CCL14 and marker genes of immune cells in GEPIA.

	Gene markers	Cancer		Normal		GTEx	
		Core	p	Core	*p*	Core	*p*
	CD8B	0.34	1.40E-11	0.5	0.00022	0.36	0.00013
T cell (general)	CD3D	0.29	1.30E-08	0.38	0.006	0.4	1.60E-05
	CD3E	0.28	7.50E-08	0.38	0.0065	0.32	6.00E-04
	CD2	0.29	9.20E-09	0.32	0.023	0.4	1.70E-05
B cell	CD19	0.069	0.18	0.12	0.43	0.22	2.10E-02
TAM	CCL2	0.28	7.00E-08	0.87	4.40E-16	0.85	0
M1 Macrophage	IRF5	0.25	1.30E-06	0.37	0.0088	0.19	4.50E-02
Dendritic cell	BDCA-1/CD1C	0.16	0.016	0.24	0.088	-0.03	7.60E-01
	BDCA-4/NRP1	0.43	0	0.36	0.01	0.44	1.50E-06
	T-bet/TBX21	0.23	9.60E-06	0.41	0.0028	0.34	2.60E-04
Th1	STAT4	0.12	0.021	0.37	0.008	0.19	5.00E-02
Tfh	CXCR5	0.39	4.70E-15	0.22	0.13	0.14	1.60E-01
T cell exhaustion	CTLA4	0.29	1.20E-08	0.42	0.0024	0.53	2.10E-09

*p < 0.05, **p < 0.01, ***p < 0.001.

### ATP1B3 Protein Expression and Prognosis in HCC

**Figure 7:**
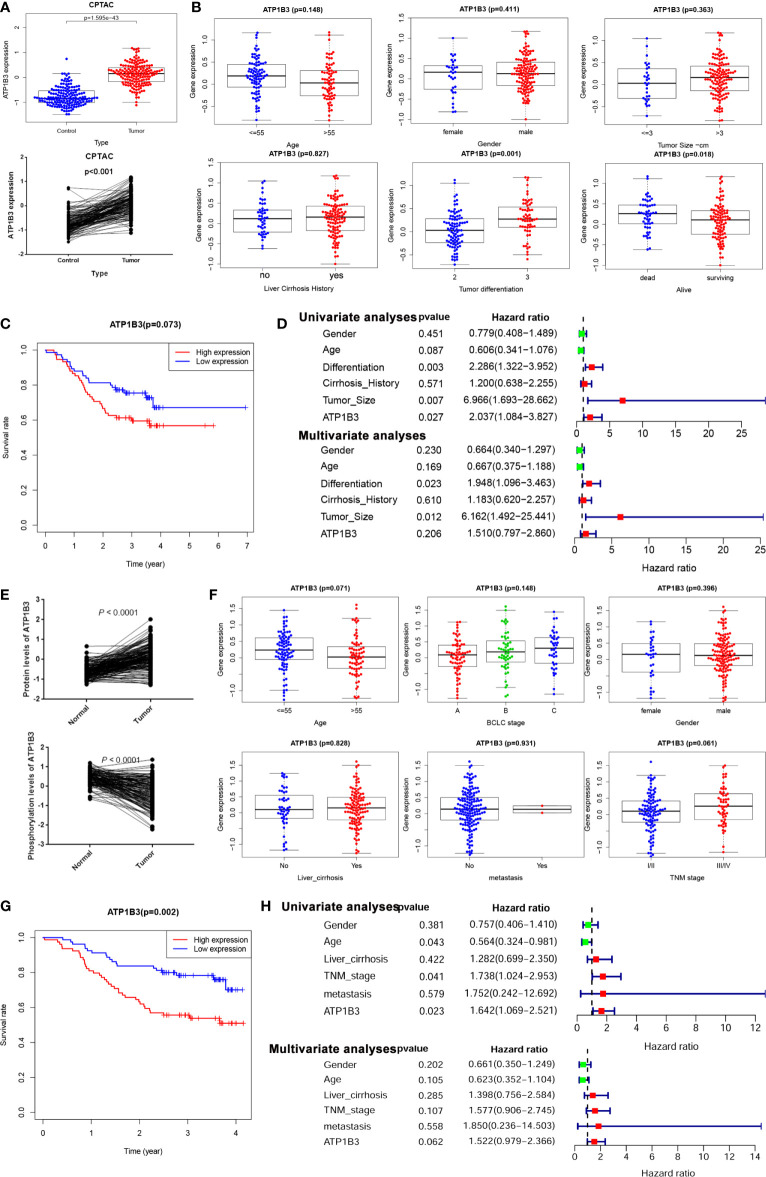
ATP1B3 protein expression and prognosis in HCC. **(A)** ATP1B3 protein levels in HCC using CPTAC proteomics database. **(B)** The association between ATP1B3 protein expression and clinical features in HCC using CPTAC proteomics database. **(C)** The survival curves of OS with high/low ATP1B3 in CPTAC HCC cohorts. **(D)** Univariate and multivariate analysis revealed the relationship between ATP1B3 and the clinical factors with OS of HCC in the CPTAC database. **(E)** The protein level and the phosphorylation level of ATP1B3 in the proteomics and phosphor-proteomics data. **(F)** The association between ATP1B3 protein expression and clinical features in the proteomics and phosphor-proteomics data. **(G)** The survival curves of OS with high/low ATP1B3 in the proteomics and phosphor-proteomics data. **(H)** Univariate and multivariate analysis revealed the relationship between ATP1B3 and the clinical factors with OS of HCC in the proteomics and phosphor-proteomics data.

To further confirm the function of ATP1B3 in HCC, we analyzed the expression and prognosis of ATP1B3 protein levels using the CPTAC proteomics database. The ATP1B3 protein level was elevated in tumor tissue compared to normal tissues (**Figure 7A**), and its’ expression was associated with the high differentiated tumor and medical history of liver cirrhosis (**Figure 7B**). HCC patients with high-expressed ATP1B3 showed poor OS (*p* =0.07) (**Figure 7C**). Moreover, the univariate analysis proved that ATP1B3, tumor size, and differentiation were significantly associated with OS in HCC, and multivariate analysis showed that tumor size and differentiation were independent factors of OS in HCC using the CPTAC database (**Figure 7D**). The relationship between ATP1B3 and 50 top negative/positive co-expressed genes were confirmed using the CPTAC database in **Figures S6** and **S7**. The correlation between ATP1B3 and immune gene was also confirmed using the CPTAC database in **Figure S8**. Moreover, the proteomics and phospho-proteomics levels of ATP1B3 of 316 HCC patients were analyzed using Gao’s data (29). As shown in **Figure 7E**, the protein level of ATP1B3 was elevated and the phosphorylation of ATP1B3 was downregulated in tumor tissue compared to paratumor tissues. And ATP1B3 protein level was associated with TNM stage (p= 0.06) (**Figure 7F**). HCC patients with high-expressed ATP1B3 shows worse OS (P=0.002) (**Figure 7G**). Moreover, the univariate analysis proved that ATP1B3, TNM, and age were significantly associated with OS in HCC, and multivariate analysis showed ATP1B3 was an independent factor of OS in HCC (**Figure 7H**).

### ATP1B3 Related Potential Drug in HCC

**Figure 8:**
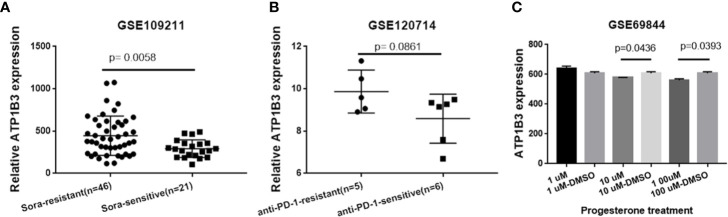
ATP1B3 related potential drug in HCC. **(A)** ATP1B3 expression in sorafenib-resistant/-sensitive HCC patients. **(B)** ATP1B3 expression in anti-PD1 immunotherapy-resistant/-sensitive HCC patients. **(C)** The GSE69844 dataset revealed that Progesterone could reduce ATP1B3 expression in HepaRG cells.

Drug sensitivity plays a crucial role in HCC treatment. We next analyzed the correlation of ATP1B3 expression to sorafenib-therapy and PD-1 immunotherapy using GSE109211 and GSE120714 database. We found that HCC patients with sorafenib-resistant have higher ATP1B3 expression compared to HCC patients with sorafenib-sensitive (**Figure 8**). However, no significant difference in ATP1B3 expression was observed between with/without PD-1 immunotherapy in HCC patients (**Figure 8B**). To further investigated the potential drug for HCC patients with high ATP1B3 expression, we analyzed the role of 34 chemicals on ATP1B3 expression using GSE69844 (**Table S9**). We found that 10 μM and 100 μM Progesterone could slightly reduce ATP1B3 expression in HepaRG cells (**Figure 8C**). These results demonstrate that Progesterone may be an expected drug for the treatment of HCC patients with high-expressed ATP1B3. This needs to be further confirmed by experiments.

### ATP1B3 Expression Is Increased in the HCC Cells and HCC Tissues

**Figure 9:**
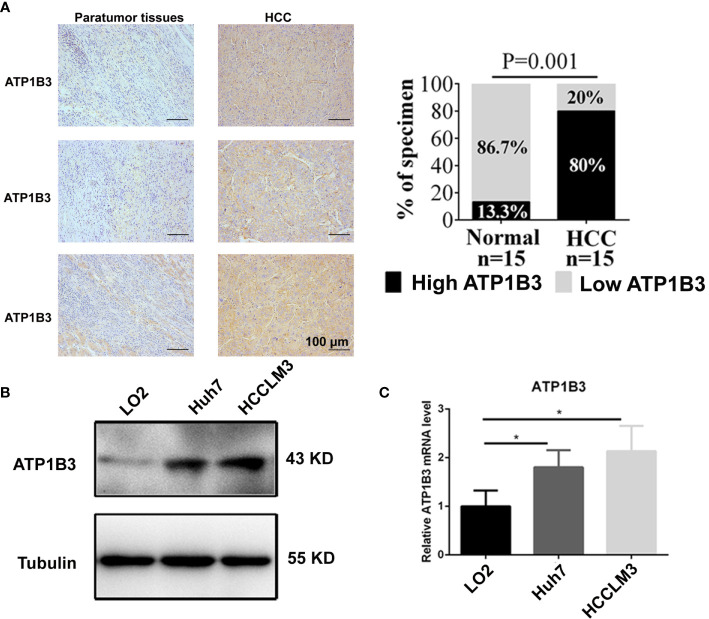
ATP1B3 expression in the HCC cells and HCC tissues. **(A)** The protein levels of ATP1B3 in HCC tissues and paratumor tissues were detected by IHC. Scale bars= 100 μm. **(B)** The protein expression level of ATP1B3 in HCC cells and normal liver cell were detected by Western Blot. **(C)** The mRNA expression level of ATP1B3 in HCC cells and normal liver cell were detected by qPCR. *p < 0.05.

We further confirmed the expression of ATP1B3 in HCC cells and HCC tissues using qPCR, western blot and IHC. As shown in **Figure 9A**, ATP1B3 is upregulated in HCC tissues compared with paratumor tissues. Similarly, compared with human normal liver cells (LO2), both protein expression and mRNA expression levels of ATP1B3 were upregulated in HCC cells (Hhu7 and HCCLM3) (**Figures 9B, C**).

### Silenced ATP1B3 Represses HCC Cell Proliferation, Migration and Induces HCC Cell Apoptosis

**Figure 10:**
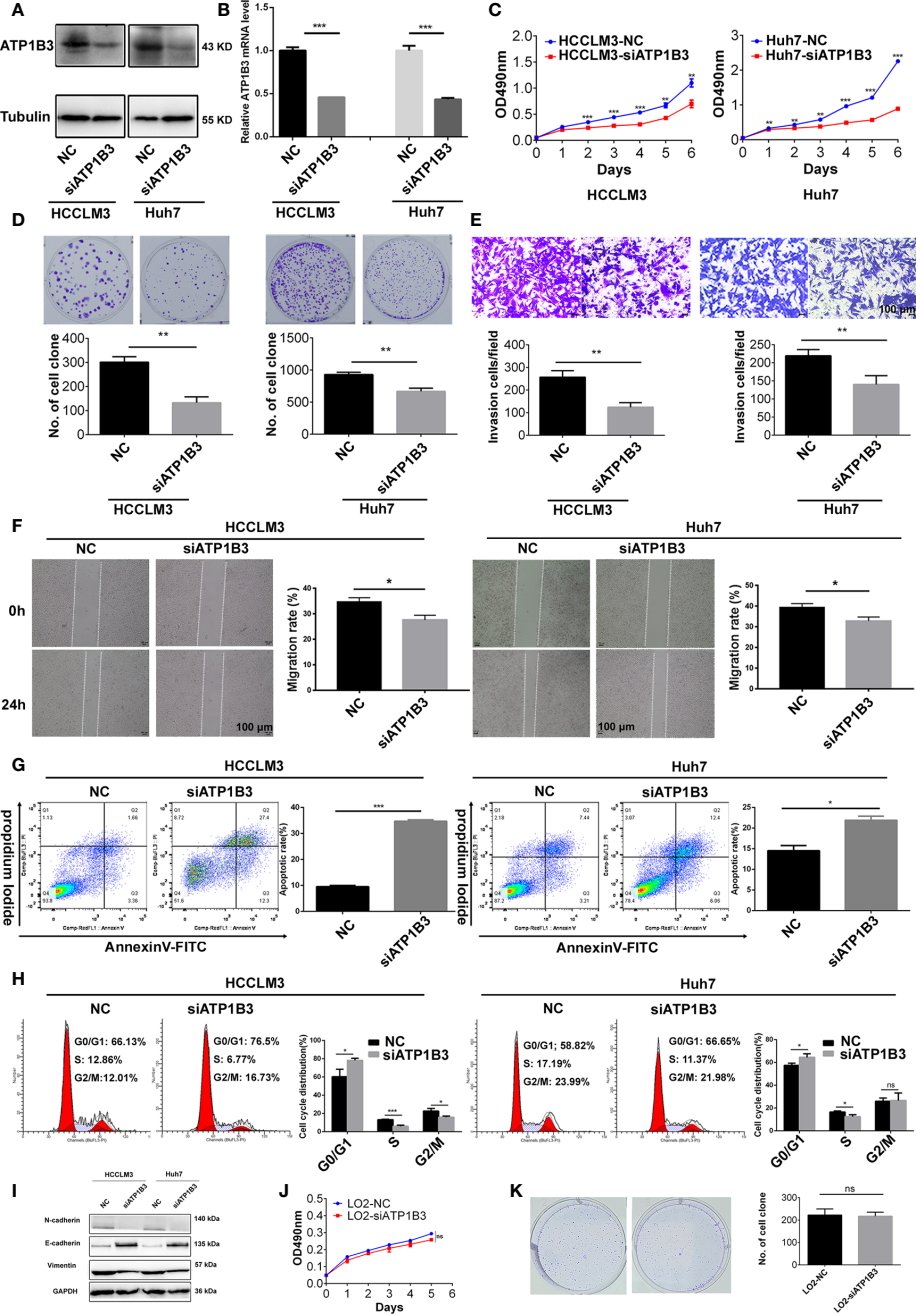
ATP1B3 promotes HCC cell proliferation, migration and inhibits HCC cell apoptosis. Western Blot **(A)** and qPCR **(B)** showed that the expression of ATP1B3 were silenced by siRNA in Hhu7 and HCCLM3, respectively. MTT **(C)** and plate clone formation **(D)** analysis revealed the cell proliferation regulated by ATP1B3. Transwell migration **(E)** and scratch wound healing **(F)** assay revealed the migration ability regulated by ATP1B3. Apoptosis **(G)** and Cell cycle **(H)** assay revealed the regulation of ATP1B3 on cell apoptosis and cell cycle using flow cytometry. **(I)** The EMT markers in ATP1B3 silenced HCC cells. The effects of ATP1B3 on cell proliferation in LO2 by MTT **(J)** and plate clone formation **(K)**. Scale bars= 100 μm, *p < 0.05, **p < 0.01, ***p < 0.001.

To investigate the role of ATP1B3 in HCC, we transfected ATP1B3 siRNA (Hhu7-siATP1B3 and HCCLM3-siATP1B3) into Hhu7 and HCCLM3 cells to knockdown ATP1B3 expression (**Figures 10A, B**), and then analyzed the effects of silenced ATP1B3 on HCC cells proliferation, migration, invasion and cycle, apoptosis. MTT and plate clone formation assay suggested that silenced ATP1B3 significantly inhibited HCC cells proliferation (**Figures 10C, D**). Transwell migration assay and wound healing assay suggested that silenced ATP1B3 significantly inhibited HCC cells migration (**Figures 10E, F**). Flow analysis suggested that silenced ATP1B3 induced HCC cells apoptosis (**Figure 10G**) and blocked cell cycle in G0/G1 phase (**Figure 10H**). Moreover, we detected the EMT markers in ATP1B3 silenced HCC cells by western blot. The results showed that silenced ATP1B3 significantly upregulated E-cadherin expression, and downregulated N-cadherin and vimentin expression. These results proved that ATP1B3 promoted EMT in HCC (**Figure 10I**). Moreover, we have tried to detect the effects of ATP1B3 on cell proliferation in LO2 by MTT (**Figure 10J**) and plate clone formation (**Figure 10K**). We found that ATP1B3 silencing had no significant effect on the proliferation of healthy liver cells. In conclusion, these results proved that ATP1B3 could promote the tumorigenicity of HCC.

## Discussion

Na^+^/K^+^-ATPase (NKA) is a multifunctional transmembrane protein that plays a crucial role in cell adhesion (14), cell movement (43), cell proliferation and apoptosis (8), and signal transduction (44). Emerging studies have shown the abnormal expression (6) and the prognosis of NKA in various cancers (9). However, the clinical relevance of NKA in HCC remains limited. In this paper, multiple public databases are used for the first time to comprehensively analyze the expression of NKA subunits in HCC and its correlation with HCC prognosis and reveal its possible mechanism in HCC.

NKA was reported to be dysregulated in multiple cancers (14). For example, NKA α1 subunit (ATP1A1) is upregulated in non-small cell lung cancer (NSCLC) (45), esophageal squamous cell carcinoma (ESCC) (46), renal cell carcinoma (47), glioma (48), but downregulated in prostate cancer (49). NKA β1 subunit (ATP1B1) is downregulated in human epithelial cancer cells (50–52). A few studies report the abnormal expression of NKA in HCC. For example, Shibuya et al. (53) and Li et al. (54) pointed out that ATP1A3 overexpression in HCC is related to the antitumor activity of bufalin. It can be used as a therapeutic target for bufalin. L Zhuang et al. (4) showed that ATP1A1 was upregulated in HCC, and its function as an oncogene by promoting proliferation and migration of HCC cells. Whereas the potential prognostic role of NKA in HCC remains unclear. Consistent with previous study, we found that ATP1A1 and ATP1A3 were upregulated in HCC from TCGA database. Moreover, ATP1B3 were also significantly upregulated in HCC with logFC > 1 and *p* < 0.01 using TCGA, ICGC, and GEO datasets. The prognostic analysis revealed that ATP1B3 was an independent factor for the OS of HCC based on transcriptomic data from TCGA, ICGC, and GEO.

ATP1B3 encodes the β3 subunit of NKA and regulates cell adhesion (55). The study has displayed that ATP1B3 expression was increased in gastric cancer tissues and was closely related to related to gastric cancer patients’ clinical characteristics (51). Here, we found that ATP1B3 high expression was associated with clinical characteristics of HCC patients including stage and grade. Subsequently, the expression and prognosis of ATP1B3 protein in HCC were also confirmed using the CPTAC database and proteomics and phospho-proteomics data from Gao’s work. The results indicated that ATP1B3 is a useful biomarker for diagnosis and prognosis of HCC prognosis. Furthermore, we validated that ATP1B3 is increased in HCC cells and tissues. Meanwhile, we also proved that silenced ATP1B3 repressed HCC cell proliferation, migration and induced HCC cell apoptosis. In brief, these results suggest that ATP1B3 could be an oncogene and promote tumorigenicity of HCC.

To investigate the potential mechanism of ATP1B3 in HCC, we analyzed the co-expressed genes of ATP1B3. The results showed that they were mainly involved in various immune responses, simultaneously inhibiting the metabolism of steroids and fatty acids. At the same time, the ATP1B3 expression was positively related to kinase expression, including LCK and LYN, which have been reported to play a crucial part in regulating B cell receptor signaling (52, 56). As previously reported that NKA regulates Src family kinase activity (including FYN and LYN) (57). Recruitment of NKA-LYN complex in macrophages promotes atherosclerosis (12). The NKA α-1/Src complex activates a variety of pro-inflammatory factors/chemokines and mediates pro-inflammatory effects (58). Pieces of evidence have proven the involvement of NKA in the inflammatory response (59). Therefore, we speculated that ATP1B3 might be involved in the immune regulation of HCC.

Immune infiltration is a significant factor in the tumor microenvironment, which plays a crucial part in the development and prognosis of tumors (60). Various immune cells contributed to the immune microenvironment of HCC including macrophages, neutrophil, dendritic cell, adaptive immune CD4+, CD8+ T-lymphocytes, and NK cells (61). Studies showed that infiltrated macrophages were polarized M2-TAM (tumor-associated macrophages), which act as immune suppressor cells and lead to reduction and exhaustion of CD8+T cells in HCC (62). Tregs were proved to be increased in HCC and impede immune surveillance (63). Despite the effect of NKA on tumor immunity has not been widely reported, it is known that knockdown of NKA α1 in macrophages can inhibit cardiotonic steroid (CTS)-induced macrophage infiltration and the accumulation of immune cells *in vivo* (64). We found that ATP1B3 was significantly correlated with tumor purity and B cell infiltration, CD8^+^ T infiltration, CD4^+^ T infiltration, Macrophage infiltration, Neutrophil infiltration, and Dendritic cell infiltration. Also, ATP1B3 and CD8^+^ T cells were found to be independent factors of HCC. In addition, we also found that ATP1B3 expression was positively correlated with the makers of CD8+ T, T cell, B cell, TAM, M1 Macrophage, DCs, Th1, Tfh, and T cell exhaustion. These immune cells are regulated by various cytokines and chemokines in the tumor environment, leading to different functions (65). Our research shows that the expression of ATP1B3 is positively correlated with IL10, IL22, IL34, and negatively correlated with IL27. IL10 was reported to inhibit the cytotoxicity of NK cells through the STAT3 signaling pathway, thereby promoting the recurrence and metastasis of HCC (66). In addition, IL22 is highly expressed in HCC and is related to the growth and malignancy of HCC tumors (67). IL-34 promotes the proliferation and migration of HCC through CSF1-R and CD138 (68). In addition, the DC-derived cytokine IL27 can exert anti-tumor activity by activating NK cells (69). These results imply that ATP1B3 may involve in immune infiltration by regulating immune-related cytokine in HCC.

Basing on the potential therapeutic and prognostic role of ATP1B3 on HCC patients, we analyzed the drug sensitivity of HCC patients with different expressed ATP1B3. Our result revealed that HCC patients with sorafenib-resistant have higher ATP1B3 expression compared to HCC patients with sorafenib-sensitive, suggesting that ATP1B expression is associated with sorafenib-resistant in HCC patients. Subsequently, 34 chemicals analysis results showed that 10 μM and 100 μM Progesterone slightly reduced ATP1B3 expression in HepaRG cells, indicating that Progesterone may be a combined drug strategy for sorafenib in the treatment of HCC. Moreover, studies showed that Na/K-ATPase is a target for anticancer drugs perillyl alcohol (POH), and Cardioprotection drug DRRSAb, indicating their potential therapeutic effect for HCC (11, 70). However, the treatment effect of these drugs for HCC has yet to be proved.

## Conclusions

In summary, our results indicate that ATP1B3 is upregulated and promote the tumorigenicity of HCC, and it is also an independent prognostic biomarker for the diagnosis of HCC with a potential immunomodulatory role, providing a novel prognostic biomarker and potential therapeutic target for HCC.

## Data Availability Statement

The original contributions presented in the study are included in the article/**Supplementary Material**. Further inquiries can be directed to the corresponding author.

## Ethics Statement

The studies involving human participants were reviewed and approved by The ethics committee of Xiangya Hospital, Central South University. Written informed consent for participation was not required for this study in accordance with the national legislation and the institutional requirements.

## Author Contributions

Conceptualization: YZ and SL. Methodology: SL, SC and XP. Investigation: SL and XP. Writing – Original Draft: YX and SL. Writing – Review and Editing: YZ, SL and RC. Funding Acquisition: YZ, RC and XP. All authors contributed to the article and approved the submitted version.

## Funding

This work was supported by the National Natural Science Foundation of China (81703149 and 81874251). This work was supported by the National Natural Sciences Foundation of Hunan province (2020JJ5950 and 2019JJ50417).

## Conflict of Interest

The authors declare that the research was conducted in the absence of any commercial or financial relationships that could be construed as a potential conflict of interest.
